# Participation and performance trends in multistage ultramarathons—the ‘Marathon des Sables’ 2003–2012

**DOI:** 10.1186/2046-7648-1-13

**Published:** 2012-12-01

**Authors:** Christoph Knoth, Beat Knechtle, Christoph Alexander Rüst, Thomas Rosemann, Romuald Lepers

**Affiliations:** 1 Department of Orthopaedics and Traumatology, Kantonsspital St. Gallen, St. Gallen, Switzerland; 2 Institute of General Practice and for Health Services Research, University of Zurich, Zurich, Switzerland; 3 Gesundheitszentrum St. Gallen, Vadianstrasse 26, St. Gallen 9001, Switzerland; 4 INSERM U1093, Faculty of Sport Sciences, University of Burgundy, Dijon, France

**Keywords:** Ultramarathon, Performance trends, Nationality, Gender difference

## Abstract

**Background:**

The purpose of this study was to investigate participation and performance changes in the multistage ultramarathon ‘Marathon des Sables’ from 2003 to 2012.

**Methods:**

Participation and performance trends in the four- or six-stage running event covering approximately 250 km were analyzed with special emphasis on the nationality and age of the athletes. The relations between gender, age, and nationality of finishers and performance were investigated using regression analyses and analysis of variance.

**Results:**

Between 2003 and 2012, a number of 7,275 athletes with 938 women (12.9%) and 6,337 men (87.1%) finished the Marathon des Sables. The finisher rate in both women (*r*^2^ = 0.62) and men (*r*^2^ = 0.60) increased across years (*p* < 0.01). Men were significantly (*p* < 0.01) faster than women for overall finishers (5.9 ± 1.6 km·h^−1^ versus 5.1 ± 1.3 km·h^−1^) and for the top three finishers (12.2 ± 0.4 km·h^−1^ versus 8.3 ± 0.6 km·h^−1^). The gender difference in running speed of the top three athletes decreased (*r*^2^ = 0.72; *p* < 0.01) from 39.5% in 2003 to 24.1% in 2012 with a mean gender difference of 31.7 ± 2.0%. In men, Moroccans won nine of ten competitions, and one edition was won by a Jordanian athlete. In women, eight races were won by Europeans (France five, Luxembourg two, and Spain one, respectively), and two events were won by Moroccan runners.

**Conclusions:**

The finisher rate in the Marathon des Sables increased this last decade. Men were significantly faster than women with a higher gender difference in performance compared to previous reports. Social or cultural inhibitions may determine the outcome in this event. Future studies need to investigate participation trends regarding nationalities and socioeconomic background, as well as the motivation to compete in ultramarathons.

## Background

Participation in endurance and ultra-endurance events such as running [1-3], triathlon [4,5], and cycling [6,7] is of high popularity. There is an increasing number of ultramarathons, defined as a running race with distance longer than 42 km in every continent [8-10], and more and more athletes from every age and gender participate in these races [1,2,11]. In ultramarathon running, single-stage and multistage races are offered. A single-stage event, such as the ‘Western States 100 Mile Endurance Run’ [1,2,10], is a non-stop race. In contrast, multistage ultramarathons such as the ‘Deutschlandlauf’ [12,13] take place over a certain number of days with recovery time overnight [13]. Among the multistage ultramarathons, one of the most challenging events is the ‘Marathon des Sables’, a multistage ultramarathon in the Moroccan desert with temperatures up to 49°C [14].

Gender differences in endurance performance have been of great interest in the last decades [2,15]. Women run approximately 10–30% slower compared to men [2,16,17]. Hoffman [15] reported no difference in performance between women and men in 80- and 161-km trail ultramarathons when matched for performance in a 50-km trail ultramarathon. However, in anecdotic reports, women were able to outrun men in ultramarathons [13,17]. A reason for the gender difference might be the higher body fat in women compared to men [18,19], and as core temperature rises, women might therefore not cope with heat as well as men [20]. Other studies suggested that slower runners may have a stronger heat impact on their running performance, which could not be demonstrated in women [21-25].

Previous studies [26-31] suggested that performance in running seemed also to be related to the athletes' origin. Most running races from middle-distance to marathon were dominated by African runners [26]. Several studies reported that East African runners from Kenya [26], Eritrea [27], and Ethiopia [28-30] dominated in long-distance running such as marathon runs worldwide, although the genetic or physiological proof is still missing [31-33]. Demographic characteristics and social and environmental factors such as where they live, which tribe they belong to, and how far the athletes had to run to school seemed to play an important role in the East African runners' performance level and participation in running competitions [26,33]. Onywera et al. [26] reported that East African competitors were very selective in choosing competitions, and a major factor for their participation was the prize money offered in competitions.

To date, little is known about the participation and performance in both single- and multistage ultramarathons regarding the aspect of the nationality of athletes. One study investigated the participation trends concerning nationality or cultural background in ultra-endurance athletes such as triathletes [34]. The authors reported that mainly Europeans competed and won in Double Iron ultra-triathlons. These findings were confirmed for longer distances such as the Triple Iron ultra-triathlon [35] and the Deca Iron ultra-triathlon [36].

The aim of the present study was to investigate the participation and performance trends in the Marathon des Sables in the desert of Morocco from 2003 to 2012. We hypothesized, firstly, that there would be an increase in participation for both sexes, secondly, that runners originating from Northern Africa would dominate the race, and, thirdly, that male ultramarathoners would be faster than female ultramarathoners.

## Methods

All finishers in the Marathon des Sables between 2003 and 2012 were analyzed regarding gender, running speed, and nationality. The data set from this study was obtained from DUV ultramarathon statistics [37] and the race website [14]. This study was approved by the institutional review board of St. Gallen, Switzerland, with waiver of the requirement for informed consent given that the study involved the analysis of publicly available data.

### The race

The Marathon des Sables is considered to be one of the most challenging ultra-running events worldwide [14]. Organized since 1986 by Frenchman Patrik Bauer, the race takes place in the Moroccan desert where multiple stages of different lengths have to be completed within 7 days. There is a four- and a six-stage setup of the course varying every year. The stages are between 20 and 40 km, and one single stage is approximately 80 km. The runners are supported with daily water rations and a tent to sleep at night but have to carry their own nutrition of at least 2,000 kcal and daily needs such as sleeping bags in backpacks. The stages and the total number of kilometers run differ every year. The course leads the participants through dry river beds, over sand dunes, and stony and rocky surfaces at temperatures up to 40°C. The number of contestants from all over the world is restricted to approximately 800 participants every year.

### Data analysis

In total, data were available from 7,275 athletes, consisting of 938 women and 6,337 men. The 7,275 athletes originated from 74 different countries and participated at least once in the Marathon des Sables. The running speed and gender difference in performance of the top three overall male and female athletes were analyzed for each year. For the aspects of nationality, such as development of running speed per country and year or difference in running speed between different countries, only the countries providing at least five finishers per year in at least nine out of the ten regarded years were considered. For women, athletes from France and Great Britain fulfilled these criteria. For men, athletes from France, Great Britain, Spain, Italy, Germany, USA, Morocco, and Switzerland were considered. From all these countries, the both running speeds of the annual top three women and annual top three men were analyzed. In women, the two nationalities that could be included had a total of 547 athletes and thus 58.3% of all female finishers. In men, the eight countries that fulfilled the inclusion criteria provided a total of 5,337 athletes and thus 84.2% of all male finishers. Since the number of stages and the total race distance varied within these years, we calculated running speed to express running performance of the athletes.

The gender difference in percent was calculated using the following formula: (Running speed [men] − Running speed [women]) / Running speed [men] × 100. In order to increase the readability of the data, the gender difference was transformed to absolute values. As a last step, we analyzed for the eight above-mentioned countries the coherence of development in population, gross national income per head, and number of finisher.

### Statistical analysis

In order to increase the reliability of data analyses, each set of data was tested for normal distribution as well as for homogeneity of variances in advance of statistical analyses. Normal distribution was tested using a D’Agostino and Pearson omnibus normality test, and homogeneity of variances was tested using a Levene's test in cases of two groups and with a Bartlett's test in cases of more than two groups. To find significant changes in the development of running speed, participation, and gender across years, linear regression was used. Correlation analyses were performed using Pearson's correlation in cases of normal distributed data and Spearman's correlation in cases of not normal distributed data. Statistical analyses were performed using IBM SPSS Statistics (Version 19, IBM SPSS, Chicago, IL, USA) and GraphPad Prism (Version 5, GraphPad Software, LA Jolla, CA, USA). Significance was accepted at *p* < 0.05 (two-sided for *t* tests). Data in the text are given as mean ± standard deviation.

## Results

### Participation trends

**Figure 1:**
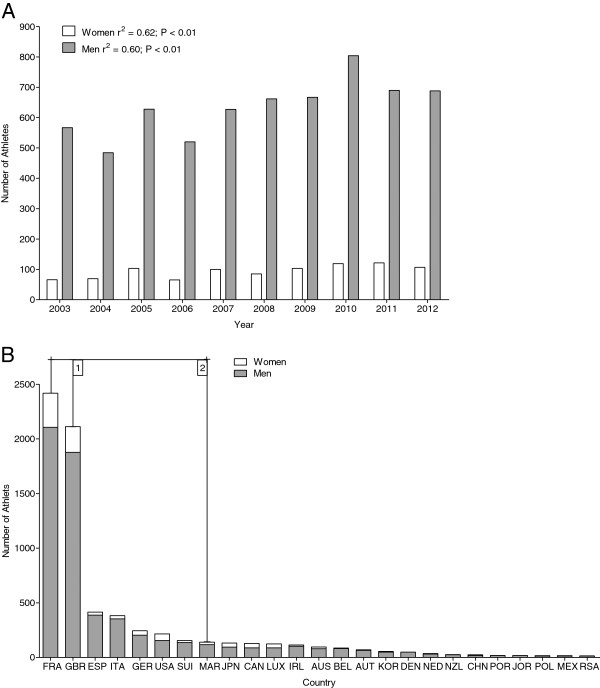
**Total number of male and female finishers (A) and number of female and male finishers in the Marathon des Sables per country (B).** Countries providing five or more finishers per year and gender are indicated with *1* for women and with *2* for men, respectively. The *frame* indicates the 25 countries with the total highest number of finishers including women and men. *FRA* France, *GBR* Great Britain, *ESP* Spain, *ITA* Italy, *GER* Germany, *USA* United States of America, *SUI* Switzerland, *MAR* Morocco, *JPN* Japan, *CAN* Canada, *LUX* Luxembourg, *IRL* Ireland, *AUS* Australia, *BEL* Belgium, *AUT* Austria, *KOR* Korea, *DEN* Denmark, *NED* Netherlands, *NZL* New Zealand, *CHN* China, *POR* Portugal, *JOR* Jordan, *POL* Poland, *MEX* Mexico, *RSA* Republic of South Africa.

Between 2003 and 2012, a total of 938 women and 6,337 men finished in the Marathon des Sables during that period, leading to a mean number of 104 female (12.8%) and 704 male (87.1%) finishers per year. Women (*r*^2^ = 0.62) and men (*r*^2^ = 0.60) showed a significant (*p* < 0.01) increase in the number of finishers (Figure 1A). More than half of the competitors of both genders (women 58.3%, men 62.9%) originated from Europe, mainly from France (women 33.2%, men 33.2%) and Great Britain (women 25.1%, men 29.7%) (Figure 1B).

### Overall performances

**Figure 2:**
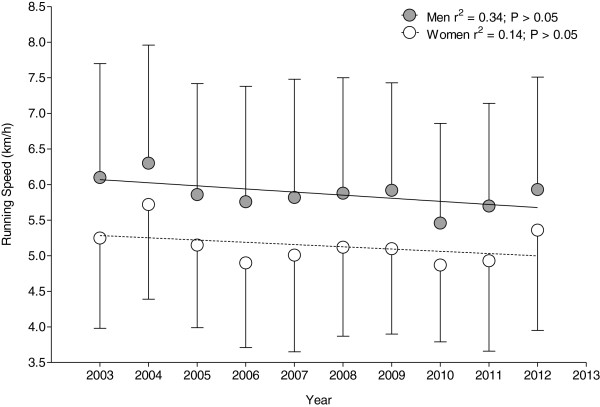
Annual running speed for women and men overall.

On average, overall men were running significantly faster than overall women (*p* < 0.01). Men were running at 5.9 ± 1.6 km·h^−1^ and women at 5.1 ± 1.3 km·h^−1^ with no change in running speed across years (*p* > 0.05) (Figure 2).

### Running performance and gender difference of the top three males and females

**Figure 3:**
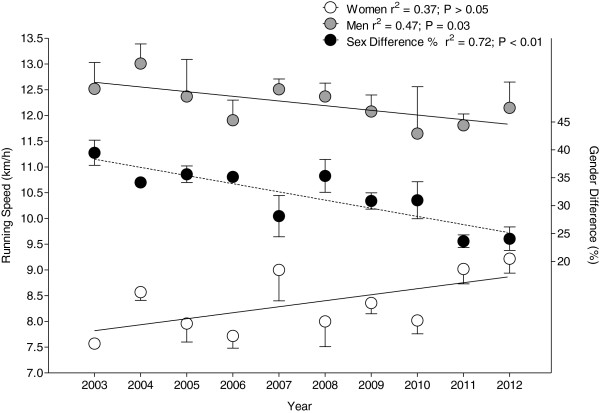
Change in running speed of the annual top three women and annual top three men and gender difference in performance across years.

During the studied time period, the top three men became slower (*r*^2^ = 0.47; *p* = 0.03) with a mean running speed of 12.5 ± 0.5 km·h^−1^ in 2003 to 12.1 ± 0.5 km·h^−1^ in 2012 (Figure 3). The top three women showed no change in running speed across years with a mean running speed of 8.3 ± 0.3 km·h^−1^ (*r*^2^ = 0.37; *p* > 0.05). The gender difference in running speed of the top three athletes decreased (*r*^2^ = 0.72; *p* < 0.01) from 39.5% in 2003 to 24.1% in 2012 with a mean gender difference of 31.7 ± 2.0% across years.

### Running speed of the top three athletes per nation and year

**Figure 4:**
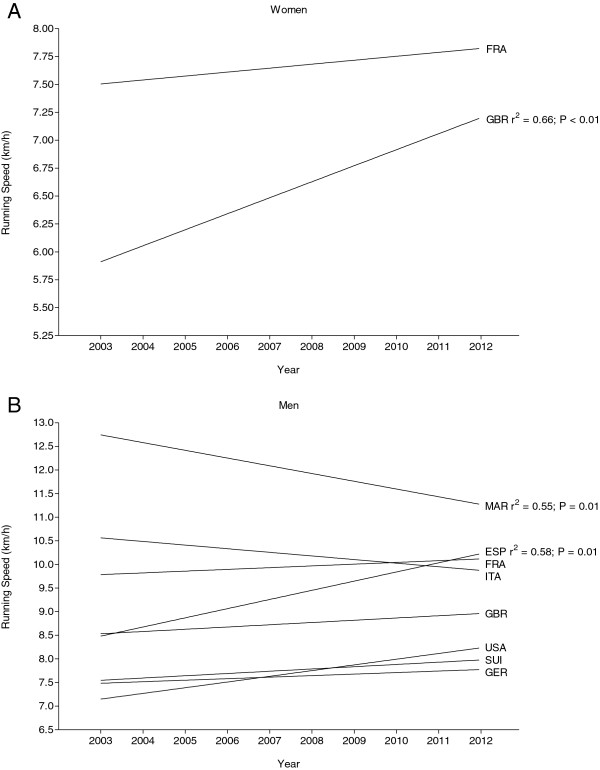
**Change in running speed in the annual top three athletes per nation during the 2003–2012 time period for women (A) and men (B).***FRA* France, *GBR* Great Britain, *ESP* Spain, *ITA* Italy, *MAR* Morocco. In case of a significant change in running speed over time, *r*^2^ and as *p* value are inserted.

**Figure 5:**
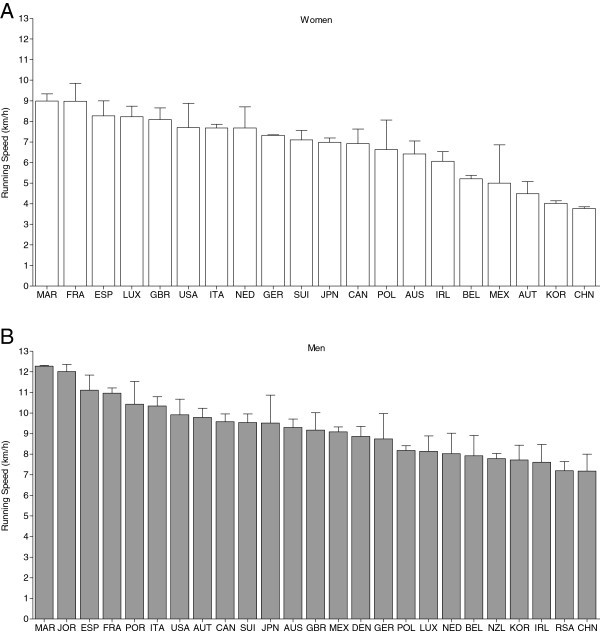
**Running speed of the overall top three female (A) and male (B) athletes per country.** The *frame* indicates the 25 countries with the total (women and men) highest number of finishers. *FRA* France, *GBR* Great Britain, *ESP* Spain, *ITA* Italy, *JOR* Jordan, *GER* Germany, *USA* United States of America, *SUI* Switzerland, *MAR* Morocco, *JPN* Japan, *CAN* Canada, *LUX* Luxembourg, *IRL* Ireland, *AUS* Australia, *BEL* Belgium, *AUT* Austria, *KOR* Korea, *DEN* Denmark, *NED* Netherlands, *NZL* New Zealand, *CHN* China, *POR* Portugal, *POL* Poland, *MEX* Mexico, *RSA* Republic of South Africa.

Figure 4 presents the trend of running speeds by the origin of the athletes for women (Figure 4A) and men (Figure 4B). For women, the annual top three British runners improved their running speed from 6.2 ± 0.6 km·h^−1^ in 2003 to 7.7 ± 1.0 km·h^−1^ in 2012 (*r*^2^ = 0.66; *p* < 0.01). For French women, running speed showed no changes (*p* > 0.05). For men (Figure 4B), the annual top three Spanish runners improved their running speed from 8.7 ± 0.4 km·h^−1^ in 2003 to 10.2 ± 0.7 km·h^−1^ in 2012 (*r*^2^ = 0.58; *p* < 0.01). Moroccan runners became slower across years with a mean running speed of 12.5 ± 0.5 km·h^−1^ in 2003 to 11.5 ± 0.8 km·h^−1^ in 2012 (*r*^2^ = 0.55; *p* < 0.01). Top three runners from France, Italy, Great Britain, USA, Switzerland, and Germany showed no changes in running speed across years (*p* > 0.05). Figure 5 presents the mean running speed of the ever fastest three runners per country for women (Figure 5A) and men (Figure 5B). For women, the top three athletes from Morocco achieved the fastest running speed (9.0 ± 0.3 km·h^−1^), followed by the top three athletes from France (9.0 ± 0.9 km·h^−1^), and the top three athletes from Spain (8.3 ± 0.7 km·h^−1^). For men, the fastest running speed ever was achieved by the top three runners from Morocco (12.3 ± 0.3 km·h^−1^), Jordan (12.0 ± 0.3 km·h^−1^), and Spain (11.1 ± 0.7 km·h^−1^). In men, Moroccans won nine of ten competitions, and one edition was won by a Jordanian. In women, Europeans (France five, Luxembourg two, and Spain one) won most of the events, and two events were won by Moroccan runners.

### The aspect of age

**Figure 6:**
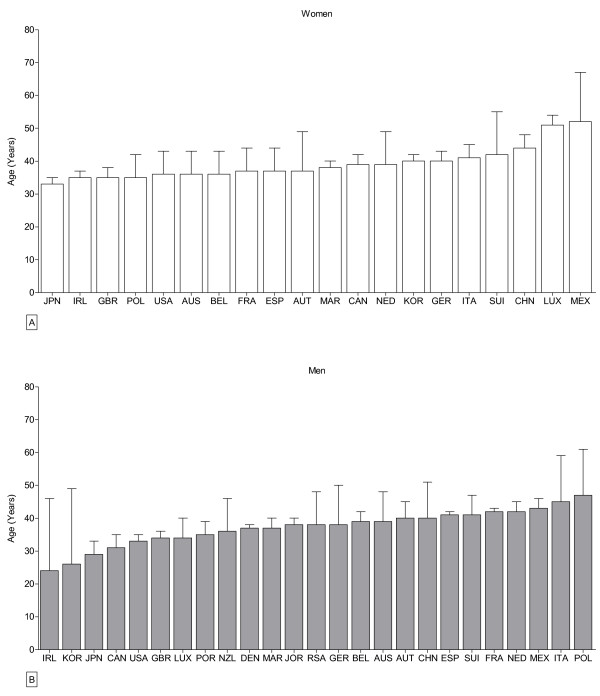
**Age of the overall top three female (A) and male athletes (B) per country. ***FRA* France, *GBR* Great Britain, *ESP* Spain, *ITA* Italy, *GER* Germany, *USA* United States of America, *SUI* Switzerland, *MAR* Morocco, *JPN* Japan, *JOR* Jordan, *CAN* Canada, *LUX* Luxembourg, *IRL* Ireland, *AUS* Australia, *BEL* Belgium, *AUT* Austria, *KOR* Korea, *DEN* Denmark, *NED* Netherlands, *NZL* New Zealand, *CHN* China, *POR* Portugal, *POL* Poland, *MEX* Mexico, *RSA* Republic of South Africa.

In women, the mean age of the overall top three finishers was 39 ± 7 years. Japanese women were the youngest participants with 33 ± 2 years of age in average, and Mexican women are the oldest competitors with 52 ± 15 years of age in average (Figure 6A). For the fastest athletes, Moroccan women were 38 ± 2 years, French women 37 ± 2 years, and Spanish women 37 ± 7 years old. In men (Figure 6B), the overall average was 37 ± 8 years of age. Irish men were the youngest with 24 ± 22 years, followed by runners from Korea (26 ± 23 years), and Japan (29 ± 4 years). Polish athletes were the oldest runners with 47 ± 14 years in average. For the fastest men, Moroccan runners were 37 ± 3 years; Jordan runners were 38 ± 2 years, and Spanish runners were 41 ± 1 years old.

**Figure 7:**
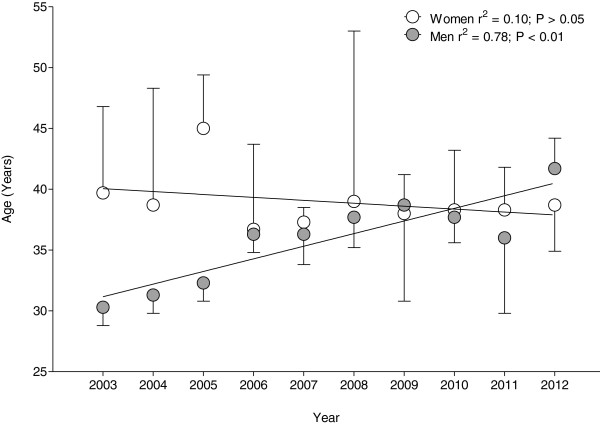
Change in the age of peak performance of the annual top three women and men across years.

Considering the changes in the age of the annual top three runners across years (Figure 7), the age of peak performance remained unchanged for women at 39.0 ± 6.3 years (*r*^2^ = 0.10; *p* > 0.05). For men, however, the age of the fastest running speed increased from 30.3 ± 1.5 years in 2003 to 41.7 ± 2.5 years in 2012 (*r*^2^ = 0.78; *p* < 0.01).

## Discussion

This is the first study to describe the participation and performance trends in a multistage ultramarathon in extreme environment such as the Marathon des Sables. The main results were (1) a significant increase in male and female finishers this last decade, (2) most finishers in both sexes originated from Europe, and (3) men were significantly faster for overall and in the top three compared to women with a higher gender difference as reported in previous studies.

### Increasing participation across years

A first important finding was the significant increase in the number of finishers for both women and men. When the Marathon des Sables started in 1986, only 23 runners participated and finished, whereas in 2012, already 795 runners finished the race. The present findings confirm the increase in participation in ultra-endurance running races as previously described for 100-km ultramarathoners [38]. Similarly, an analysis of participation in 161-km ultramarathons in North America showed that the number of both competitors and competitions significantly increased over the last decades [1,2].

### Most finishers in the Marathon des Sables originated from Europe

A second important finding was that half of the finishers of both genders originated from Europe, mainly from France and Great Britain. By comparison, Rüst et al. [34] reported that most finishers in a Double Iron ultra-triathlon originated from Europe. We assume that there is a higher interest of competitors out of ‘first world countries’ [39] to compete in extreme endurance challenges. With a high inscription fee and a low prize money of 5,000 euros for the overall winner, the Marathon des Sables is expensive for participants. The inscription fee for the Marathon des Sables is approximately 2,800 euros [14]. In contrast, participation in the ‘Boston Marathon’ costs US$180 for residents and US$200 for international competitors [40], whereas the prize money is approximately ten times higher [41]. We assume that European competitors, as the largest group of finishers in both sexes, have the economic background to take part in most ultramarathon events worldwide; competitors from second or third world countries are not able to pay for the dispenses and are reliant on sponsors or prize money to be able to compete all over the world - a reason that East African runners do not dominate in economically less interesting ultra-endurance events. The cultural background might be of importance as well: France has a long history and a close relationship with Morocco, and the Marathon des Sables is organized by a Frenchman. This could be one of the reasons why many French runners compete in the Marathon des Sables.

### Participation trends, population growth, and gross national income per capita

We investigated the participation trends, population growth and gross national income per head of the eight countries that could be analyzed from 2003 to 2011 (Table 1). Although all countries showed a significant growth of their population (*p* < 0.01) between 2003 and 2011 and a significant increase in income per head (*p* < 0.01), only Great Britain (*r*^2^ = 0.65), Spain (*r*^2^ = 0.97), and Switzerland (*r*^2^ = 0.74) showed a significant increase in finishers relative to the change in population (*p* < 0.01). Spain and Switzerland showed also a significant correlation between the increase in gross national income per head and the increase in participants (*p* < 0.01). Most probably, the economic situation of an athlete decides whether he will be able to participate in the Marathon des Sables. Although Moroccan men won almost every competition, in women, European runners won eight out of ten competitions; only two events were won by female Moroccan runners. No East African runner even participated in the Marathon des Sables in the last 10 years. According to Onywera [26,33], the social background of African runners was very important for their success and that their participation in endurance events was triggered by economic reasons and social background. Onywera et al. [26] showed that East African competitors were very selective in choosing competitions, and a major factor for participation was the prize money available in competitions. Recent studies suggested a mental advantage of African runners because of the nimbus of winning [29] and the psychological atmosphere that could have significant consequences on performance [42], because no genetic advantages have been found. East African runners differed largely in their demographic characteristics. Factors, such as how far the athletes had to run to school and their socioeconomic background, seemed to play a great role in later success [26,33]. According to the results of Onywera's findings [26], there would be a lower participation of East African runners in economically less interesting events such as the Marathon des Sables. Furthermore, we expected wealthier competitors with less interest in earning money and more interest in the challenge of ultramarathon participation. Hofmann and Fogard [43] showed that participants in a 161-km ultramarathon were largely well-educated, middle-aged, and married men who rarely missed work due to illness or injury, generally used vitamins and/or supplements, and maintained appropriate body mass with aging. Their findings may confirm our investigations as we found a significant increase in the gross national income per head from 2003 to 2011 in the eight countries included in the analysis. Also, a significant correlation between participation and income per head for Spanish and Swiss competitors could be seen (Table 1, [44-46]). Similar to Hoffman and Fogard's work [43], most competitors were middle-aged with an average age of 39 years in women and 37 years in men. This demonstrates that older runners with the financial background and the will to prove themselves instead of younger runners with economic interests participate in ultramarathons such as the Marathon des Sables.

**Table 1:** Increase in finishers, population and GNI per capita, PPP, and correlation between finishers and population and participants and GNI per capita 2003–2011

	**Finishers**				**Population (in 1,000)**				**Finishers / Population**						**Finishers / Income**	

	**2003**	**2011**	***r***^***2***^	***p***	**2003**	**2011**	***r***^***2***^	***p***	**2003**	**2011**	***r***^***2***^	***p***	***r***^***2***^	***p***	***r***^***2***^	***p***
FRA	240	230	0.09	>0.05	59,765	63,126	0.96	<0.01	27,460	35,860	0.92	<0.01	0.32	>0.05	0.26	>0.05
GBR	180	228	0.65	<0.01	59,554	62,417	1.0	<0.01	30,250	36,970	0.74	<0.01	0.82	<0.01	0.65	>0.05
ESP	9	76	0.97	<0.01	41,875	46,452	0.96	<0.01	24,470	31,930	0.80	<0.01	0.92	<0.01	0.79	<0.01
ITA	37	46	0.0	>0.05	57,604	60,789	1.0	<0.01	27,080	32,350	0.77	<0.01	0.05	>0.05	0.30	>0.05
GER	33	17	0.09	>0.05	82,533	82,163	0.74	<0.01	28,110	40,170	0.94	<0.01	0.02	>0.05	0.27	>0.05
USA	20	20	0.31	>0.05	290,810	313,085	1.0	<0.01	38,400	48,490	0.81	<0.01	0.54	>0.05	0.41	>0.05
SUI	8	24	0.74	<0.01	7,284	7,702	0.91	<0.01	36,100	50,900	0.96	<0.01	0.78	<0.01	0.85	<0.01
MAR	11	13	0.00	>0.05	30,088	32,644	0.92	<0.01	3,060	4,910	1.0	<0.01	0.07	>0.05	0.06	>0.05

Increase in finishers per country, population per country (in thousands) [44,45] and gross national income per capita, purchasing power parity (PPP) (current international dollar) [46], correlation between finishers and population and participants and gross national income per capita from 2003 to 2011. *FRA* France, *GBR* Great Britain, *ESP* Spain, *ITA* Italy, *GER* Germany, *USA* United States of America, *SUI* Switzerland, *MAR* Morocco.

### Who is winning, and why are there gender differences?

The fastest performance was attained by Moroccan competitors, winning nine of ten editions from 2003 to 2012, although most male finishers in the Marathon des Sables originated from France and Great Britain. We expected the same outcome in women, but only two out of ten competitions were won by Moroccans; eight races were won by Europeans (France five, Luxembourg two, and Spain one). Moroccan women achieved the fastest running speeds, followed by French and Spanish women. The dominance of European females might be due to cultural and religious inhibitions. In Islamic countries, females seem to participate less in competitive sports; therefore, fewer females from Morocco might be competing in the Marathon des Sables [47]. Between 2003 and 2012, only 24 Moroccan women and one Pakistani woman competed, which is only approximately 3% of the female competitors. Another reason might be the high inscription fees and costs to participate in such an event. Most probably, North African women were not as wealthy as European competitors, and the financial backgrounds as well as social or religious reasons were limiting female participation in the Marathon des Sables.

### Running performance and gender difference

A third important finding was that the top three men became slower across years, whereas the top three women showed no change in running speed across years. The gender difference in running speed of the top three athletes decreased (*r*^2^ = 0.72; *p* < 0.01) from 39.5% in 2003 to 24.1% in 2012 with a mean gender difference of 31.7 ± 2.0% across years. In endurance performance, the gender difference varied largely from 10% to 30% [15,17]. According to previous findings, running performance might be impaired by a higher core temperature [21]. This could be due to higher body fat levels in females [19,48] as well as the stronger heat impact on slower runners, although the effect could not be shown for women [22-25].

An interesting finding was that men became slower and that women showed no change in running speed across years. The decrease in running speed in men could be due to the increase in age in men. The increasing popularity of the Marathon des Sables and the concurrent increase of amateur competitors of all levels might be a reason for the decrease in overall running speed in men. Most probably, more men seek the adventure and thrill a competition like the Marathon des Sables provides and that the competitors in the beginning of the Marathon des Sables were prepared better and in a better shape overall as there were only few competitors and the event was not as popular as today [14]. For the unchanged performance in women, the increase in participation in women in ultramarathon might be a potential reason [1]. With a general increase in participation of women, more concurrence occurs, and the level of running speed may rise. Another aspect might be that the running speed rises with the popularity of the Marathon des Sables as more women were participating and training more specifically to compete in this event. In men, Spanish runners were the only ones who were becoming faster across years. Most probably, athletes from countries improving their performance might have had special training programs or national support for their matter [49]. Another reason might be that competing and winning in ultramarathons are more prestigious in certain countries. Because of the similarity of the Mediterranean climates with hot summer temperatures in southern Europe, Spanish competitors might adapt to the heat and extreme conditions easier. Interestingly, Moroccans became slower across years although they would be able to train in the same environment and might therefore have an advantage.

### Master runners dominate the Marathon des Sables

The fastest women achieved their best performances at the age between 35 and 40 years (Moroccan women with 38 ± 2 years, French women with 37 ± 2 years, and Spanish women with 37 ± 7 years). Also for men, the fastest runners were between 35 and 40 years old (Moroccan men with 37 ± 3 years, Jordanian men with 38 ± 2 years, and Spanish men with 41 ± 1 years). In addition, the age of the top three runners in 2012 was 41.7 ± 2.5 years for men and 38.7 ± 3.8 years for women. Reaburn and Dascombe [50] described that master athletes were typically older than 35 years of age, were systematically trained, and competed in organized forms of sport specifically designed for older adults. This was due to the observed age-related decline in endurance performance in different sport disciplines such as running, orienteering, rowing, and swimming. Following the definition of Reaburn and Dascombe [50], the top runners in the Marathon des Sables were all master runners. However, the present findings suggest that ultramarathoners do not fall within this definition of master athletes after the age of 35 years since the annual fastest runners in recent years and the fastest runners by country were between 35 and 40 years old.

### Limitations

This study is limited because physiological [24], nutritional [51] or environmental [23], and medical [52] influences were not considered. Additionally, the different course and changing numbers of stages might play a role in running speed. These factors may as well have an influence on the outcome of a multistage ultra-endurance race. A questionnaire for income, job, and social background to investigate the socioeconomic influence on participation is missing. The influence of training and equipment [53] might well be a substantial factor for performance, and there might be a correlation between wealth of a nation and the equipment and nutrition the competitors can afford. That may be of importance especially in competitions under extreme circumstances, and further investigations should be done on these parameters.

## Conclusions

The number in finishers in the Marathon des Sables increased in the last decade. Most finishers originated from Europe, mainly from France and Great Britain. Moroccan men were the fastest runners, winning nine of ten competitions between 2003 and 2012. We assume that the socioeconomic and religious background plays an important role in participation and running speed in the Marathon des Sables. Men were significantly faster than women, and the mean gender difference of 31.7 ± 2.0% in the top three was rather high compared with previous studies. Moreover, the fastest runners were master athletes. Further studies need to investigate the gender differences in running speed in very hot climates and participation trends concerning nationalities and socioeconomic background, by questionnaire related to participation in ultra-endurance events. The age of peak performance in other ultra-endurance races needs to be determined, and the definition of master athlete as athletes aged > 35 years and competing in sports specifically designed for older adults needs to be questioned.

## Competing interests

The authors declare that they have no competing interests.

## Authors’ contributions

All authors—CK, BK, CAR, TR, and RL—designed the study. The manuscript was written by all authors. All authors read and approved the final manuscript.
